# COVID-19 Pandemic-Delayed Diagnosis and Treatment of Atypical Neuroleptic Malignant Syndrome in a Violent Forensics Patient With Intellectual Disability and Treatment-Resistant Schizophrenia

**DOI:** 10.7759/cureus.41866

**Published:** 2023-07-14

**Authors:** Mian Zarshawn H Jan, Patricia Figgs, Gerard Gallucci, Romona Bacchus

**Affiliations:** 1 Psychiatry, The Delaware Psychiatric Center, New Castle, USA; 2 Psychiatry and Behavioral Sciences, The Delaware Psychiatric Center, New Castle, USA

**Keywords:** prison inmates, intellectual disability (id), schizophrenia, clinical psychiatry, covid 19, neuroleptic malignant syndrome (nms)

## Abstract

This case report highlights an episode of neuroleptic malignant syndrome (NMS) in a forensic psychiatry inpatient unit and how the coronavirus disease (COVID) pandemic, as well as, an atypical presentation of NMS delayed diagnosis and treatment of a patient, which could have been fatal. NMS and atypical NMS manifest typically after the use of anti-psychotics during the first two weeks of initiation of treatment. COVID can mimic many of the initial symptoms of NMS such as changes in mental status, fever, and, at times, dysautonomia. This case will try and highlight why this crossover of symptoms and the forensic environment made diagnosis and treatment in this particular case more difficult.

## Introduction

Neuroleptic malignant syndrome (NMS) is defined as “a life-threatening neurologic emergency associated with the use of neuroleptic agents and characterized by a distinctive clinical syndrome of mental status change, rigidity, fever, and dysautonomia,” the incidence of which is thought to be around 0.02-3% in patients taking neuroleptics [1-3]. Dysautonomia leads to mortality, which today is considered to occur in around 10-20% of cases [2,4]. NMS is most likely to occur in the first two weeks of treatment; however, there have been cases of patients on the same medication without dose change for many years experiencing NMS [5]. The tetrad of mental status changes, muscular rigidity, fever, and dysautonomia occur in around 97% of patients over the course of one to three days, with mental status changes as the most common initial symptom. In atypical NMS, however, only two of the tetrad of symptoms need to be present in conjunction with an offending agent [1]. It has been noted that rigidity can be mild or absent in these atypical cases [6]. Laboratory testing can be invaluable, especially in the setting of atypical NMS, when symptoms alone may not be enough to guide clinical decision-making. Basic lab values include elevated creatinine kinase (CK) from 1,000 to 100,000 U/L, leukocytosis with WBC counts of 10,000 to 40,000 units/nL, elevations in liver enzymes, electrolyte abnormalities, myoglobinuria and proteinuria, elevated eosinophils, and low serum iron [1,3,5,7-9]. This case report intends to highlight the significant symptom overlap between COVID and NMS, and how this led to a delayed diagnosis of NMS, which could have been fatal for this patient.

## Case presentation

Mr. H. is a 36-year-old African American male, with a history of schizophrenia, intellectual developmental disability (IDD), and phencyclidine use disorder, who was first admitted to a forensic psychiatry hospital in November 2017. He was deemed Not Guilty by Reason of Insanity (NGRI) for aggravated menacing. His records from previous hospital admissions at other hospitals surmised a long history of treatment-resistant schizophrenia with frequent violent outbursts, which he had been treated for with multiple high-dose medication trials, including risperidone, valproic acid, and chlorpromazine over the past 20 years. During his time in the forensic unit between the years 2017 and 2020, he had been trialed on several anti-psychotic agents with varying success. The patient was first trialed on chlorpromazine 100 mg PO TID for three months, followed by olanzapine 20 mg PO qdaily for nine months, neither of which alone was able to properly control his psychosis. His then current regimen as of August 2020, included haloperidol 5 mg PO qAM, haloperidol 10 mg PO qHS, and olanzapine 10 mg PO qHS, along with the rest of his medications listed in Table 1. At the time of his onset of NMS in August 2020, he was still residing in the level 5 inpatient forensic psychiatry hospital. The patient was seen by the treatment team on August 26 and was noted to have continued symptoms of paranoia with auditory hallucinations, very poor insight, frequent violent altercations, and great difficulty articulating his needs and symptoms given his IDD and likely cognitive decline from chronic phencyclidine (PCP) use. The treatment team agreed that down-tapering his olanzapine and starting clozapine was appropriate at that time. Olanzapine was lowered from 10 mg to 5 mg and clozapine was started at 12.5 mg BID. A complete blood count (CBC) and comprehensive metabolic panel (CMP) were within normal limits.

**Table 1:** Medication list as of 8/23/2020

Psychiatric Medication	All other Medications
Trazodone 50 mg PO qHS	Vitamin D3 1000 mg PO qdaily
Fluoxetine HCL 20 mg PO qdaily	Omeprazole 40 mg PO qdaily
Benztropine 0.5 mg PO qHS	Lisinopril 40 mg PO qdaily
Haloperidol Dec 200 mg IM q4wks	Metoprolol Tartrate 50 mg PO BID
Olanzapine 10 mg PO qHS	Nicotine Lozenges 2 mg PO QID PRN
Haloperidol 5 mg PO qAM	
Haloperidol 10 mg PO qHS	

Seven days later, the patient complained of cough, dyspnea, loss of taste, malaise, and subjective fever with chills. As the COVID pandemic was at its height, the top of the differential diagnosis for any patient with fever was COVID. The patient was isolated and polymerase chain reaction (PCR) tested, as rapid tests were not available. The following day, he complained of neck stiffness, which was initially assumed to be part of his viral syndrome. When his COVID test came back negative, it was a shock to the treatment team, who then reconvened and discussed the possibility of other conditions at play for this patient. Given his difficulties with verbal communication and his clear psychosis, altered mental status was difficult to ascertain, but the neck stiffness and fever were mildly concerning for NMS, and a CBC, creatine kinase (CK), lactate dehydrogenase (LDH), and CMP were drawn. Clozapine was stopped, but the olanzapine and oral haloperidol were not stopped immediately because the concern for NMS was relatively low, and previous attempts to down taper olanzapine were met with a resurgence of violent paranoia that wreaked havoc on the whole unit. The decision to continue anti-psychotic treatment was a difficult one, as NMS has the potential for lethality but keeping the patient untreated could have led to the resurgence of paranoia in an almost nonverbal aggressive adult male and potential harm to himself or others. Haloperidol decanoate 200 mg had been administered just two weeks prior and was certainly part of the equation as well. The next day, the patient spiked a fever with an elevated heart rate. He was given menthol lozenges, guaifenesin, and acetaminophen 650 mg PO q8hrs and he was placed on q2hr vitals, with unspecified viral syndrome still higher on the differential than NMS. His temperature returned to normal with acetaminophen; however, his cough and neck pain persisted. Results from the NMS blood draw returned over 48 hours later, significantly delayed due to pandemic-related staffing and supply shortages, and showed an elevated CK of 6,103 U/L.

Mr. H was sent to the local ER, now with emergent concern for NMS whose diagnosis and treatment had been delayed by almost 48 hours. Labs were repeated and were significant for elevated neutrophils and hyponatremia with an updated CK of over 13,000 U/L. Blood and WBCs were found in his urine, but the fever resolved and did not return. A comprehensive list of the patient's lab results is listed in Table 2 and Table 3. His oxygen saturation never dropped below 93% on room air, and a chest X-ray and EKG were within normal limits. He was immediately taken off all antipsychotics and assumed to have NMS but was put back on olanzapine 5 mg by the ER secondary to his immense risk of violence. He was started on dantrolene IV 110 mg/2.2 ml IV q6hrs and normal saline 0.9% IV at 1000 mL. He was seen by the consult-liaison psychiatry team on the day after admission from the ER, and they promptly discontinued olanzapine and started divalproex sodium 500 mg BID to control the risk of violence. He continued to complain of a cough, neck pain, and stiffness, as well as dark urine.

**Table 2:** Pertinent comprehensive metabolic panel and complete blood count levels trended CK = creatine kinase, ALT = alanine transaminase AST = aspartate aminotransferase Text in bold represents an abnormal value.

Date	CK (U/L)	Platelets (U/nL)	Absolute Eosinophils (U/nL)	Absolute Neutrophils (U/nL)	ALT (IU/L)	AST (IU/L)	Creatinine (mg/dL)
8/26/2020		259	0.3	2.2	28	22	0.96
9/3/2020	6103	276	0.5	5.3			
9/4/2020	10646	322	0.5	5.7	41	41	1.01
9/5/2020	13474	330	0.4	5.5			
9/6/2020	18232	408	0.4	6.7			1.05
9/7/2020	18700				76	92	0.95
9/8/2020	43900	487	0.6	7.1	111	156	0.9
9/9/2020		419	0.6	7.8	108	138	0.75
9/10/2020	28000	397	0.6	7.3	138	109	0.77
9/11/2020	21500	396	0.5	7.7	132	124	0.75
9/12/2020	10600	391	0.6	5.9	138	109	0.71
9/13/2020	5898						0.75
9/14/2020	3386	405	0.5	3.7			0.71
9/15/2020	1923						0.68
9/16/2020	1456	443					0.72
9/17/2020	1814	443	0.3	3.7			0.69
9/18/2020		422	0.3	2.7			0.7
9/30/2020		292	0.3	2.2	26	20	0.8
10/7/2020	91						

**Table 3:** Pertinent comprehensive metabolic panel and complete blood count levels trended (continued) BUN = blood urea nitrogen Text in bold represents an abnormal value.

Date	BUN (mg/dL)	Albumin (g/dL)	Sodium (mmol/L)	Calcium (mg/dL)	Random Glucose (mg/dL)
8/26/2020	12	4.4	138	9.7	87
9/3/2020					
9/4/2020	11	4	134	9.3	132
9/5/2020			131		128
9/6/2020	8		137	8.2	124
9/7/2020	6	3.3	140	8.4	117
9/8/2020	7	3.4	138	8.4	135
9/9/2020	6	3	141	8.1	106
9/10/2020	8	2.9	139	8.4	102
9/11/2020	8	3.1	141	8.3	100
9/12/2020	7	3	142	8.2	93
9/13/2020	5	3.2	138	8.2	123
9/14/2020	6	3	142	8.5	91
9/15/2020	7	3	142	8.6	102
9/16/2020	5		142	8.5	
9/17/2020	9		137		
9/18/2020	11		142	8.5	
9/30/2020	8	3.8	138	9.1	122
10/7/2020					

By the third day after admission, the patient's initial symptoms had all resolved other than the cough. Lisinopril was discontinued for concern of renal failure and amlodipine 5 mg PO daily was started, as well as furosemide 20 mg/2 mL IV BID. On the same day, the CK level peaked at 43,900 U/L.

On the fifth day after admission, the IV dantrolene was changed to PO dantrium 100 mg PO QID. The patient continued to have coughing fits and a second chest X-ray was done, showing blunting of the left posterior costophrenic angle, which may have been secondary to a small pleural effusion or consolidation.

On day seven, the dantrium was decreased in coordination with decreasing CK levels, and on day 11, he was discharged back to the forensic psychiatry unit with creatinine, blood urea nitrogen (BUN), and albumin almost returned to baseline and CK rapidly decreasing. Dantrium was tapered until 9/20 when it was discontinued, 15 days after his admission to the medical hospital. He was maintained only on divalproex sodium ER 500 mg BID and PRN benzodiazepines to control his paranoia and risk of violence.

Interestingly, it was determined through record review at the medical hospital that the patient had transaminitis with profound rhabdomyolysis 14 years prior, assumed to be NMS as well. It was attributed to his risperidone, and he was treated with bromocriptine during that previous episode.

On September 30, approximately one month after the initial trial, the patient was re-trialed on clozapine 25 mg PO qHS and divalproex sodium ER 500 mg was increased from BID to TID dosing. He was also started on clonazepam 1 mg po BID due to labile mood and the risk for aggression. The patient had been off of oral haloperidol and olanzapine for 23 days at the time clozapine was restarted. He was up-titrated on clozapine by 25 mg per week until a 250 mg daily total was reached. His CK levels were checked weekly with his CBCs to monitor for a repeat episode of NMS. Mr. H. became somewhat sedated during the clozapine up-taper and the clonazepam was slowly down-tapered to counteract sedation, with good results. In February 2021, he became sedated again and divalproex sodium was slowly down-tapered, with good results. As of March 2021, Mr. H. was receiving 275 mg of clozapine a day. His auditory hallucinations have resolved, and he has not had a single violent outburst since starting clozapine.

## Discussion

Mr. H displayed only symptoms of elevated fever (102.3^o^F), dysautonomia, and mild rigidity at the onset of his NMS episode, making COVID-19 much higher on our initial differential diagnosis. At a time when COVID cases were reaching into the tens of thousands, with a high mortality rate, and where speculation continued about its spread, prevention, and best practices, it was imperative to prevent infection and keep both staff and peers safe. That thought distracted the treatment team from the true diagnosis in this case and delayed proper treatment. The inability to determine the presence of altered mental status in this already cognitively impaired patient and the relative lack of muscle stiffness represented a very atypical presentation of NMS. The patient’s symptoms of cough and fever, without other hallmark symptoms of NMS, turned out to be a red herring and caused a delay in identifying the diagnosis. This clinical decision-making delay was combined with a long lag in laboratory results returning due to staffing and supply shortages, the combination of which could have been fatal.

Another interesting piece of this case is the etiology of the patient’s episode of NMS. One possibility is that NMS developed due to the addition of clozapine to the patient’s treatment regimen. It falls neatly into the time frame for NMS, as it was started within two weeks of the NMS episode. Another possibility is that the NMS was caused by a pre-existing antipsychotic like haloperidol. The haloperidol dose was relatively high - haloperidol decanoate 200 mg IM every four weeks plus oral haloperidol 15 mg per day (he preferred to keep the oral dose despite the option of having all of his haloperidol in the decanoate shot). However, the patient was taking high-dose haloperidol for at least three years without incident. And of course, olanzapine could have been the culprit. A review of the literature suggests that clozapine is the least likely antipsychotic to cause NMS and haloperidol is the most likely [1,10]. We believe that it is likely the combination of all three of these antipsychotics that contributed to this particular episode.

The reinitiation of an antipsychotic was another high-risk aspect of this case. The current literature shows that restarting the offending agent may or may not cause a recurrence of NMS, with relapse rates between 10% and 90%, which is not helpful in real-world clinical decision-making [1,4,7,11]. Guidelines for antipsychotic reinitiation support waiting at least two weeks before restarting a neuroleptic agent, using lower potency antipsychotics, using low doses, with slow up-titration, avoiding the concomitant use of lithium, maintaining hydration, and carefully monitoring for a return of NMS symptoms. There was no question about the need to restart antipsychotic medication in this particular patient for reasons already listed, but which one? Balancing the risk that clozapine was the offending agent of this NMS episode versus the body of literature supporting the use of clozapine in post-NMS antipsychotic reinitiation, we decided to start clozapine low and go slow. We were highly alert to the fact that the COVID pandemic might complicate our decisions, as it had before, and we worked closely with nursing staff in monitoring vitals and any symptoms that came up. We monitored labs and vitals vigilantly. Ultimately, this strategy proved successful.

## Conclusions

This case describes a patient in an inpatient forensic facility with severe and persistent mental illness (SPMI) who had been treated with multiple, high doses of antipsychotic medication and who developed NMS for the second time in his life. Although the patient was at very high risk for further NMS episodes, he required further antipsychotic treatment given his severe psychotic illness associated with extremely violent outbursts. The case was complicated by his atypical presentation of NMS that occurred in the setting of the COVID pandemic, which delayed his diagnosis and treatment. We count ourselves and this patient lucky and hope future clinicians can find usefulness in this case.
